# Supplementary material for: Rates of cardiovascular events among patients with moderate-to-severe atopic dermatitis in an integrated health care system: A retrospective cohort study
Source: PLoS One. 2022 Nov 17;17(11):e0277469. doi: 10.1371/journal.pone.0277469 (PMC9671329; doi:10.1371/journal.pone.0277469)
Supplement: S1 Table — AD, atopic dermatitis; ICD, International Classification of Diseases; JAK, Janus kinase; KP, Kaiser Permanente; KPNC, Kaiser Permanente Northern California; TB, tuberculosis. (PDF) [file pone.0277469.s001.pdf]

S1 Table. **Patient Exclusion Criteria.**

Patients identified with the following criteria ever (numbers 1–4) or in the 365 days before study-specific index date (numbers 5–13) or in the 6 months before study-specific index date (number 14) were excluded from the study to align with subjects enrolled in the abrocitinib AD clinical trial program:

1. Had a solid organ or autologous bone marrow transplant (ICD-9 code V42 or ICD-10 code Z94, Z48.2, Z95.3, Z95.4);
2. Documented evidence of infection with human immunodeficiency virus;
3. Infection with hepatitis C or with hepatitis B based on laboratory results;
4. Documented evidence of a cancer diagnosis(ses), excluding NMSC and cervical carcinoma in situ;
5. Active forms of other inflammatory skin disease (*eg*, psoriasis [ICD-9 code 696.1 or ICD-10 code L40.9] or systemic lupus erythematosus [ICD-9 710.0 or ICD-10 M32.9]);
6. Prior therapy with any JAK inhibitors (*ie*, tofacitinib, ruxolitinib, or baricitinib);
7. Diagnosis of TB (ICD-9 011.9 or ICD-10 A15.0);
8. Current or past condition associated with thrombocytopenia (ICD-9 287.3, 287.4, 287.5 or ICD-10 D69.3, D69.4, D69.5, D69.6) or with any coagulopathy (ICD-9 286, ICD-10 D65, D66, D67, D68);
9. History of other lymphoproliferative disorders, including Waldenstrom macroglobulinemia (ICD-9 273.3 or ICD-10 C88.0), Wiskott-Aldrich syndrome (ICD-9 279.12 or ICD-10 D82.0), pityriasis lichenoides (ICD-9 696.2 or ICD-10

L41.0-L41.1), posttransplant lymphoproliferative disorder (ICD-9 238.77 or ICD-10 D47.Z1), autoimmune lymphoproliferative syndrome (ICD-9 279.41 or ICD-10 D89.82);

10. Diagnoses of disseminated herpes simplex or zoster: herpetic septicemia, visceral herpes simplex, herpes viral hepatitis, herpesvirus encephalitis, or disseminated herpes viral disease (ICD-9 054.3, 054.5, 054.71 or ICD-10 B00.4, B00.7, B00.81);
11. Electrocardiogram results suggestive of long QT (ICD-9 426.82 or ICD-10 145.81);
12. Advanced kidney disease (defined as ICD-9 585.3 or ICD-10 N18.3), chronic kidney disease (stage IV [severe]) (ICD-9 585.4 or ICD-10 NI8.4), chronic kidney disease (stage V) (ICD-9 585.5 or ICD-10 N18.5), or end-stage renal disease (ICD-9 585.6 or ICD-10 N18.6);
13. Advanced liver disease (defined as history of ascites) (ICD-9 789.5 or ICD-10 R18), malignant ascites (ICD-9 789.51 or ICD-10 R18.0), other ascites (ICD-9 789.59 or ICD-10 R18.8), alcoholic cirrhosis of liver with ascites (ICD-9 571.2 or ICD-10 K70.31), alcoholic hepatic failure (ICD-9 571.3 or ICD-10 K70.40,K70.41), hepatic encephalopathy (ICD-9 572.2 or ICD-10 K72.90, K72.91), portal hypertension (ICD-9 572.3 or ICD-10 K76.6), hepatorenal syndrome (ICD-9 572.4, or ICD-10, K76.7), or other sequelae of chronic liver disease (ICD-9 572.8 or ICD-10 K72.10, K72.90)
14. Any infections 6 months before index date that needed hospitalization or parenteral antimicrobial therapy.

AD, atopic dermatitis; ICD, International Classification of Diseases; JAK, Janus kinase; KP, Kaiser Permanente; KPNC, Kaiser Permanente Northern California; TB, tuberculosis.
