# Supplementary material for: Rates of cardiovascular events among patients with moderate-to-severe atopic dermatitis in an integrated health care system: A retrospective cohort study
Source: PLoS One. 2022 Nov 17;17(11):e0277469. doi: 10.1371/journal.pone.0277469 (PMC9671329; doi:10.1371/journal.pone.0277469)
Supplement: S2 Table — ICD, International Classification of Diseases; KPNC, Kaiser Permanente Northern California; MACE, major adverse cardiovascular event; MedDRA, Medical Dictionary for Regulatory Activities. aAsterisks used in ICD-9 and ICD-10 codes denote that all additional codes with numbers following the number to the left of the asterisk were also included. (PDF) [file pone.0277469.s002.pdf]

S2 Table. List of Variables and Operational Definitions.

Variable	Operational definition for alignment with KPNC databases
Major adverse cardiovascular event	Composite measure of:<br>Cardiovascular death<br>Death due to acute myocardial infarction<br>Sudden cardiac death<br>Death due to heart failure<br>Death due to stroke<br>Death due to cardiovascular procedures<br>Death due to cardiovascular hemorrhage<br>Death due to other cardiovascular causes: peripheral artery disease<br>Non-fatal myocardial infarction<br><br>Non-fatal stroke of any classification, including reversible focal neurologic defects with imaging evidence of a new cerebral lesion consistent with ischemia or hemorrhage MedDRA<br><br>Fatal and non-fatal events: 10000891, 10006148, 10007522, 10008034, 10008088, 10008190, 10014498, 10019005, 10019016, 10024033, 10028596, 10028602, 10033697, 10043647, 10049768, 10051078, 10056237, 10059613, 10060839, 10060840, 10061256, 10062573, 10064961, 10066591, 10066592, 10067167, 10067347, 10067462,

Variable	Operational definition for alignment with KPNC databases
	10068621, 10068644, 10069020, 10070671, 10070754, 10071043, 10073945, 10074422, 10008120, 10055677, 10071260, and 10008119<br><br>Fatal only: 10002886, 10003173, 10003210, 10003212, 10006145, 10007522, 10007554, 10007556, 10007558, 10007559, 10007560, 10007625, 10007684, 10007686, 10007688, 10008023, 10008030, 10008076, 10008086, 10008089, 10008092, 10008111, 10008112, 10008118, 10008132, 10018985, 10022758, 10022840, 10022841, 10024119, 10024242, 10034476, 10036511, 10039163, 10039330, 10042316, 10042317, 10042364, 10042365, 10042434, 10047279, 10048380, 10048761, 10049418, 10049780, 10049993, 10050157, 10050403, 10051093, 10051328, 10052019, 10053633, 10053649, 10053949, 10055803, 10058178, 10060874, 10060874, 10060874, 10060953, 10060964, 10062585, 10062599, 10063081, 10063082, 10063083, 10063084, 10064595, 10064601, 10065441, 10065558, 10067057, 10068119, 10068230, 10069694, 10069695, 10069696, 10071716, 10072043, 10072789, 10073565, 10073681, 10075449, and 10076203<br>ICD-10: Hospitalizations and deaths coded to I21, I22, I23, I24, I46. I60, I61, I62, I63, and I64<br>Deaths coded: I71.0, I71.1, I71.3, I75.5, I71.8 and I77.2

Variable	Operational definition for alignment with KPNC databases
	Kaiser (ICD-9): 346.6, 410, 410.00, 410.01, 410.02, 410.10, 410.11, 410.12, 410.50, 410.51, 410.52, 410.60, 410.61, 410.62, 410.70, 410.71, 410.72, 410.80, 410.81, 410.82, 410.90, 410.91, 410.92, 414.12, 414.8, 423.0, 427.5, 428, 428.0, 428.1, 428.20, 428.21, 428.22, 428.23, 428.30, 428.31, 428.32, 428.33, 428.40, 428.41, 428.42, 428.43, 428.9, 429.79, 430, 430-438, 431, 432, 432.0, 432.1, 432.9, 433, 433.00, 433.1, 433.30, 433.31, 433.80, 433.81, 433.90, 433.91, 434.00, 434.01, 434.10, 434.11, 434.90, 434.91, 437.3, 437.8, 440, 440.0, 440.2, 440.20, 440.21, 440.22, 440.23, 440.24, 440.29, 440.30, 440.31, 440.32, 440.4, 440.8, 440.9, 440-449, 441.1, 441.3, 441.5, 441.6, 442.81, 443.21, 443.22, 443.24, 443.9, 444, 444.01, 444.21, 447.2, 707.11, 707.12, 707.13, 707.14, 707.15, 707.19, 707.8, 779.89, 785.4, 785.51, 798.0, 798.1, 798.2, 798.9, 799.9, I60, I60-I69
Venous thrombotic events	Kaiser (ICD-9): O87.3, O22.50, I63.6, I67.6, I82 (O00-O07, O08.7; O22.-, O87.-; I63.6, I67.6; I21-I25; G08; I67.6; G95.1; K55.0-; I81; I26.-), I82.91, O22.5, O22.51, O22.52, O22.53, I82.90, I82.890, I82.431, I82.432, I82.502, I82.511, I82.512, I82.513, I82.521, I82.522, I82.523, I82.531, I82.532, I82.401, I82.402, I82.411, I82.412, I82.413, I82.433, I82.533, I82.A11, I82.A12, I82.A13, I82.A21, I82.A22, I82.A23, I82.C11, I82.C12, I82.419, I82.421, I82.422, I82.429, I82.439, I82.519, I82.529, I82.7 (Z86.718; Z79.01)<br><br>ICD-10-CM Diagnosis code: I82.7

Variable	Operational definition for alignment with KPNC databases
	Chronic embolism and thrombosis of veins of upper extremity I82.A19, I82.A29, G08 (O00-O07, O08.7; O22.5, O87.3; I67.6; G95.1), I82.403, I82.891
Deep vein thrombosis	Kaiser (ICD-9): O87.1, I82.4-, I82.5-, I82.62-. I82.72-, O22.3, I82.4-, I82.5-, I82.62-. I82.72-, O22.30, I80.20, I80.29, I82.4, I82.409, I80.291, I80.292, I80.293, I82.509, I80.2, I80.299, I82.49, I82.503, I82.59, I82.40, I82.50, I82.629, I82.721, I82.401, I82.402, I82.4Y, I82.4Y1, I82.4Y2, I82.4Y3, I82.4Z, I82.5Y, I82.5Y3, I82.5Z, I80.209, I82.491, I82.492, I82.493, I82.499, I82.4Y9, I82.4Z1, I82.4Z2, I82.4Z3, I82.4Z9, I82.501, I82.502, I82.591, I82.592, I82.593, I82.599, I82.5Y1, I82.5Y2, I82.5Y9, I82.5Z1, I82.623, I82.723, I82.403, I82.621, I82.622, I82.722, I82.729, I82.5, I82.62, I82.72, I80.201, I80.202, I80.203, I80.13, I82.419, I82.519, O22.31, O22.32, O22.33, I82.429, I82.439, I82.529, I82.A29, I82.423, I82.521, I82.522, I82.523, I82.890, I82.411, I82.412, I82.413, I82.433, I82.A11, I82.A12, I82.A13, I82.A21, I82.A23, I82.C11, I82.C12, I80.1, I80.11, I80.12, I82.432, I82.511, I82.512, I82.513, I82.221, I82.891, I82.421, I82.422, I82.431, I82.531, I82.532, I82.C13, I82.C23, T81.72XA, I82.A22, I82.C19, I82.C29, I82.41, I82.51, I82.220, I82.C21, I82.C22, I80.8
Pulmonary embolism[a]	ICD-9: 415.1* and ICD-10 code: I26*

ICD, International Classification of Diseases; KPNC, Kaiser Permanente Northern California; MACE, major adverse cardiovascular event; MedDRA, Medical Dictionary for Regulatory Activities. [a]Asterisks used in ICD-9 and ICD-10 codes denote that all additional codes with numbers following the number to the left of the asterisk were also included.
