# Supplementary material for: Rates of cardiovascular events among patients with moderate-to-severe atopic dermatitis in an integrated health care system: A retrospective cohort study
Source: PLoS One. 2022 Nov 17;17(11):e0277469. doi: 10.1371/journal.pone.0277469 (PMC9671329; doi:10.1371/journal.pone.0277469)
Supplement: S3 Table — AD, atopic dermatitis; CI, confidence interval; MACE, major adverse cardiovascular event. aItems with non-overlapping confidence intervals for comparisons within the moderate AD population and within the severe AD population are shown in bold for comparisons. (PDF) [file pone.0277469.s003.pdf]

S3 Table. Incidence Rates per 1000 Person-Years of Cardiovascular Events in the Cohort of KPNC Health Plan Members (2007-2018) Aged ≥12 Years With Moderate and Severe AD by Sex, Smoking Status, and Diabetes Status.

Cardiovascular event		Incidence rates per 1000 PY (95% CI) among patients with moderate AD (N = 7158)	Incidence rates per 1000 PY (95% CI) among patients with severe AD (N = 1039)
**MACE**		2.5 (2.0–3.2)	3.2 (1.8–5.7)
Sex	Women[a]	**1.7 (1.2–2.4)**	**0.5 (0.1–3.5)**
	Men	**4.2 (3.1–5.6)**	**6.5 (3.6–11.7)**
Smoking status	Smoker	5.1 (2.6–10.2)	5.4 (0.8–38.2)
	Never smoker	1.8 (1.2–2.6)	2.1 (0.8–5.5)
	Former smoker	3.7 (2.2–6.4)	8.5 (3.2–22.4)
Diabetes status	Diabetes	**8.9 (5.5–14.3)**	16.7 (4.2–65.9)
	No diabetes	**2.1 (1.7–2.7)**	2.8 (1.5–5.2)
**Venous thrombotic event**		1.9 (1.5-2.5)	2.1 (1.1-4.2)
Sex	Women	1.9 (1.4–2.6)	1.0 (0.2–4.0)
	Men	2.1 (1.4–3.1)	3.4 (1.5–7.5)
Smoking status	Smoker	1.2 (0.3–4.9)	0
	Never smoker	1.9 (1.3–2.7)	3.1 (1.4–6.9)
	Former smoker	3.9 (2.3–6.6)	0
Diabetes status	Diabetes	**4.9 (2.6–9.1)**	7.5 (1.1–52.6)
	No diabetes	**1.7 (1.3–2.3)**	1.9 (0.9–4.0)
**Deep vein thrombosis**		1.6 (1.2–2.1)	1.6 (0.7–3.5)
Sex	Women	1.6 (1.1–2.2)	1.0 (0.2–3.9)
	Men	1.6 (1.0–2.6)	2.2 (0.8–6.0)

Smoking status	Smoker	1.2 (0.3–4.9)	0
	Never smoker	1.6 (1.1–2.4)	2.6 (1.1–6.2)
	Former smoker	3.1 (1.7–5.6)	0
Diabetes status	Diabetes	**3.9 (2.0–7.8)**	7.5 (1.1–52.6)
	No diabetes	**1.4 (1.1–1.9)**	1.4 (0.6–3.3)
**Pulmonary embolism**		0.7 (0.4-1.0)	1.1 (0.4–2.8)
Sex	Women	0.8 (0.5–1.2)	1.0 (0.2–3.9)
	Men	0.4 (0.2–1.1)	1.1 (0.3–4.5)
Smoking status	Smoker	0	5.3 (0.7–37.2)
	Never smoker	0.5 (0.2–1.0)	1.0 (0.3–4.1)
	Former smoker	1.4 (0.6–3.4)	2.0 (0.3–14.0)
Diabetes status	Diabetes	1.5 (0.5–4.5)	7.5 (1.1–53.0)
	No diabetes	0.6 (0.4–0.9)	0.8 (0.3–2.5)

AD, atopic dermatitis; CI, confidence interval; KPNC, Kaiser Permanente Northern California; MACE, major adverse cardiovascular event; PY, person-years.

[a]Items with non-overlapping confidence intervals for comparisons within the moderate AD population and within the severe AD population are shown in bold for comparisons.
